# Value of pharmacy services upon admission to an orthopedic surgery unit

**DOI:** 10.1186/s40545-021-00384-x

**Published:** 2021-12-06

**Authors:** Ahmad El Ouweini, Lamis R. Karaoui, Nibal Chamoun, Chahine Assi, Kaissar Yammine, Elsy Ramia

**Affiliations:** 1 Lebanese American University, School of Pharmacy, P.O. Box S-23, Byblos, Lebanon; 2 Lebanese American University Medical Center – Rizk Hospital (LAUMC-RH), Beirut, Lebanon; 3 Lebanese American University – School of Medicine, Byblos, Lebanon

**Keywords:** Medication reconciliation, Medication errors, Orthopedic surgery, Pharmacy services

## Abstract

**Background:**

In Lebanon, the role of the pharmacist remains underestimated in the medication reconciliation process, especially in surgical departments. This study aims to assess the impact of pharmacist-conducted medication reconciliation performed within 48 h of hospital admission to the orthopedic surgical department.

**Methods:**

This was a prospective single-arm study conducted in a tertiary-care teaching hospital in Lebanon between October 2019 and April 2020. Participants were adult inpatients hospitalized for orthopedic surgeries with ≥ 1 outpatient medications. Properly trained pharmacy resident obtained the Best Possible Medication History (BPMH) and led the reconciliation process. The primary endpoint was the number of reconciliation errors (REs) identified. Descriptive statistics were used to report participants’ responses and relevant findings. Linear regression was performed with the number of REs as a continuous dependent variable using backward method. Results were assumed to be significant when p was < 0.05.

**Results:**

The study included 100 patients with a mean age of 73.8 years, admitted for elective (54%) or emergency (46%) surgeries. Half of the study population had ≥ 5 home medications. The mean time for taking BPMH was around 8 min. A total of 110 REs were identified in 74 patient cases. The most common discrepancies consisted of medication omission (89.1%) and the most common medications involved were antihyperlipidemic agents. Twenty-four REs were judged as clinically significant, and four as serious. The most common interventions included the addition of a medication (71.9%). Most of the relayed interventions (84.5%) were accepted. The number of home medications was the only variable significantly associated with the number of REs (*β* 0.492; *p* < 0.001).

**Conclusion:**

Pharmacy-led medication reconciliation upon admission to orthopedic surgery department can reduce reconciliation errors and improve medication safety.

***Trial registration*:**

Retrospectively registered in the Lebanon Clinical Trials Registry (LBCTR2020124680).

**Supplementary Information:**

The online version contains supplementary material available at 10.1186/s40545-021-00384-x.

## Background

Medication reconciliation is the process of creating the most accurate list possible of all medications a patient is taking and comparing this list to the physician’s admission, transfer, and/or discharge orders, to provide the correct medications to the patient at all transitions of care [1]. Numerous patient safety organizations have recommended the implementation of this practice in health centers, given reports that medication reconciliation can reduce reconciliation errors (REs) by up to 75% and associated adverse drug events (ADE) by 15–18% [2]. Although medication errors have the potential to occur throughout the hospitalization process, the admission process has been shown to be particularly vulnerable [3]. Implementation of an established medication reconciliation process upon hospital admission may reduce medication errors and ADE in hospitals by 46% and 20%, respectively [3]. Pharmacist involvement within the medication reconciliation process was also shown to improve the effectiveness of identifying and rectifying these discrepancies [2]. Surgical patients receive chronic treatments with at least one drug that is not related to the disease responsible for the surgery, and it is essential to adequately manage their medications both pre- and post-operatively [4]. During the preoperative period, discontinuation of chronic medication may compromise disease control or lead to the development of withdrawal symptoms, while the continuation of certain drugs may increase the surgical risk or interact negatively with concurrent medications such as anesthetics, causing severe reactions [4]. In a study that assessed the potential impact of medication histories obtained by pharmacists in a preoperative anesthesia clinic, pharmacists have identified and resolved clinically meaningful medications discrepancies in 61% of the patients. Hence, this has enhanced the accuracy of obtained home medications list [5]. Another study showed that when medication reconciliation was performed on cardiac surgery patients upon admission, transfer and discharge, medication discrepancies trended downwards from admission until discharge [6]. This highlights the importance of continuity of care in order to successfully complete the process of medication reconciliation [6]. Roure Nuez et al. demonstrated that reconciliation programs and perioperative drug management improve the safety of drug use in patients undergoing surgery and enhance the efficiency of the perioperative medication management system [7]. In another Spanish study that included 176 patients admitted for surgery, 55.1% of the participants had at least one RE, with a mean of 3.21 REs per patient and a maximum of 12 [8].

In Lebanon, the practice of medication reconciliation is not fully implemented in all Lebanese hospitals, and the role of the pharmacist remains underestimated [9, 10]. Only around 41% of hospital pharmacists in Lebanon reported performing medication reconciliation upon admission, transfer of care, or discharge [9]. In a study that examined the impact of pharmacy-led medication reconciliation, unintended medication discrepancies were common on hospital admission to the internal medicine services [10]. Pharmacy-led medication reconciliation upon admission, with student pharmacist involvement and physician communication helped reduce unintended discrepancies and improve medication safety [10]. The Lebanese Ministry of Public Health (MOPH), in its most recent version of hospital accreditation standards released in January 2019, recommended to perform medication reconciliation during admission and discharge, and to share the documented medication list with all the healthcare providers, pharmacy and patient [11].

To our knowledge, there are no studies in Lebanon assessing the impact of medication reconciliation performed on surgery patients. The study was conducted at the Lebanese American University Medical Center—Rizk Hospital (LAUMC-RH), a 197-bed tertiary-care teaching hospital located in Beirut, Lebanon. A well-defined policy for guiding the process of medication reconciliation did not exist in the hospital at the time of this study. Additionally, the pharmacist role in conducting medication reconciliation is limited. While there are clinical pharmacy specialists serving in cardiology, critical care, emergency medicine, infectious diseases and internal medicine departments, there are no clinical pharmacists serving on the orthopedic surgery unit.

The primary objective of this study was to assess the impact of pharmacy-led medication reconciliation performed on adult patients admitted to the orthopedic surgical department with ≥ 1 chronic medication, 24–48 h of hospital admission, measured by the incidence of REs or unintended discrepancies identified. Secondary outcomes included the number of interventions performed to resolve discrepancies and their clinical significance. The time needed for medication history, and the information sources used to complete the Best Possible Medication History (BPMH) were also reported.

## Methods

### Study design and patient recruitment

This was a prospective single-arm pilot study conducted over a 7-month period between October 2019 and April 2020 in the Orthopedic Surgery Department. The project was approved by the Institutional Review Board (IRB) of the hospital and the Lebanese American University prior to the beginning of data collection. IRB approval number: LAU.SOP.ER2. 30/Sep/2019. Accordingly, all participating patients were requested to sign a written informed consent.

Patients were prospectively identified within 48 h of hospital admission by obtaining a daily list from the pharmacy department at LAUMC-RH, excluding weekends, and confirming the list with the clinical coordinator of the orthopedic surgery department. Included patients were ≥ 18 years old, admitted for at least 48 h to the Orthopedic Surgery Department for elective or emergency surgeries, and currently taking at least one regular prescription medication. Patients were excluded if they were admitted for less than 2 days or were unable to communicate in English or Arabic.

### Intervention and data collection

A post-graduate year 1 pharmacy resident interviewed the eligible patients after obtaining their written informed consent to participate in the study. The aim of the interview was to collect and document the Best Possible Medication History (BPMH). The pharmacy resident inquired about all prescription and over-the-counter medications. He asked both open-ended and closed-ended questions to trigger the patient to remember medications that they may have forgotten to mention such as creams, ointments, inhalers, eye drops, ear drops, vitamins, and herbal or dietary supplements. To ensure complete documentation of the BPMH, the resident also noted the reported level of compliance, the last dose taken, and potential recent changes to select medication regimens. In order to obtain the BPMH, the pharmacy resident relied on more than one information source such as interviewing the family/caregiver, inspecting the medication bottles, or reviewing the patient’s previous health record available at the institution. The pharmacy resident documented all the information on the “Medication Reconciliation Data Collection Form” that was developed to guide the BPMH process and record the reconciliation findings (Additional file 1). This form included general demographic data, surgery type, total number of home medications and their indications, history of drug allergies, and results of the critical analysis of discrepancies. The different sections of this data collection form and the patient interview tips were adapted from the Medications at Transitions and Clinical Handoffs (MATCH) Toolkit for Medication Reconciliation [12]. Afterwards, the pharmacy resident compared the obtained history from the patient to the medications ordered by the physician for the patient’s current admission. When deemed necessary, the pharmacy resident intervened in the management of the patient’s medication regimens during the reconciliation process by contacting the physician and clarifying any changes that need to be implemented to the current medication regimens.

### Main outcomes and measures

The resident critically analyzed the identified discrepancies and classified them according to the MATCH toolkit: no discrepancies (i.e., one-to-one match), intended discrepancies which are appropriate discrepancies based on the patient’s comorbidities and clinical status or unintended discrepancies (considered as REs) [12]. The primary outcome consisted of the number of REs, defined as any unjustified or unintended discrepancy between the patient’s medications prior to admission/surgery, and the inpatient medication list, 24–48 h after admission. REs were described as unintended discrepancies because there was no justification based on the patient’s comorbidities and clinical condition. They included any inappropriate omission or addition of a medication, substitution of an agent within the same pharmacological class and change in dose, frequency, or route of administration. REs were then classified by type, medication category, therapeutic/pharmacological class, route of medication involved, and whether or not the discrepancy relates to a high-alert medication as per the Institute of Safe Medication Practices (ISMP) [13]. The potential severity of REs was classified using a validated scale, stratifying severity in four levels (1 = clinically insignificant, 2 = clinically significant, 3 = serious and 4 = life-threatening). Clinically insignificant RE refers to an error that would not likely cause harm, clinically significant RE refers to an error that has the potential to cause harm, and may require increased monitoring, serious RE refers to an error that has potential to cause harm and (1) likely to require additional intervention or (2) could result in prolonged hospital length of stay); and life-threatening RE refers to an error having the potential to cause death or likely lead to death without the use of life-sustaining interventions [14]. A consensus was reached by the panel of investigators for classification of all discrepancies.

Secondary outcomes included the number of pharmacy resident’s interventions performed to resolve discrepancies. The time needed for BPMH, and the information sources used to complete the BPMH were also documented.

### Data management and statistical analysis

The data collected were coded, entered into SPSS software version 26, verified for data entry errors, and analyzed. Descriptive statistics were used to report all participants’ responses. The association between categorical variables was evaluated using the Pearson Chi-squared test or Fisher’s exact test where the expected cell count < 5. Linear regression was performed with the number of REs (unintended discrepancies) as a continuous dependent variable using backward method [15]. The independent variables included age, gender, allergies, number of drugs prior to hospital admission, therapeutic class of the drug involved in the discrepancy according to the first level Anatomical Therapeutic Chemical (ATC) Classification and the type of hospital admission (elective or emergency). Variables with a *p*-value of 0.2 or less in the bivariate analysis were included in the initial model. Two-sided *p* value of < 0.05 was considered statistically significant.

## Results

**Fig. 1:**
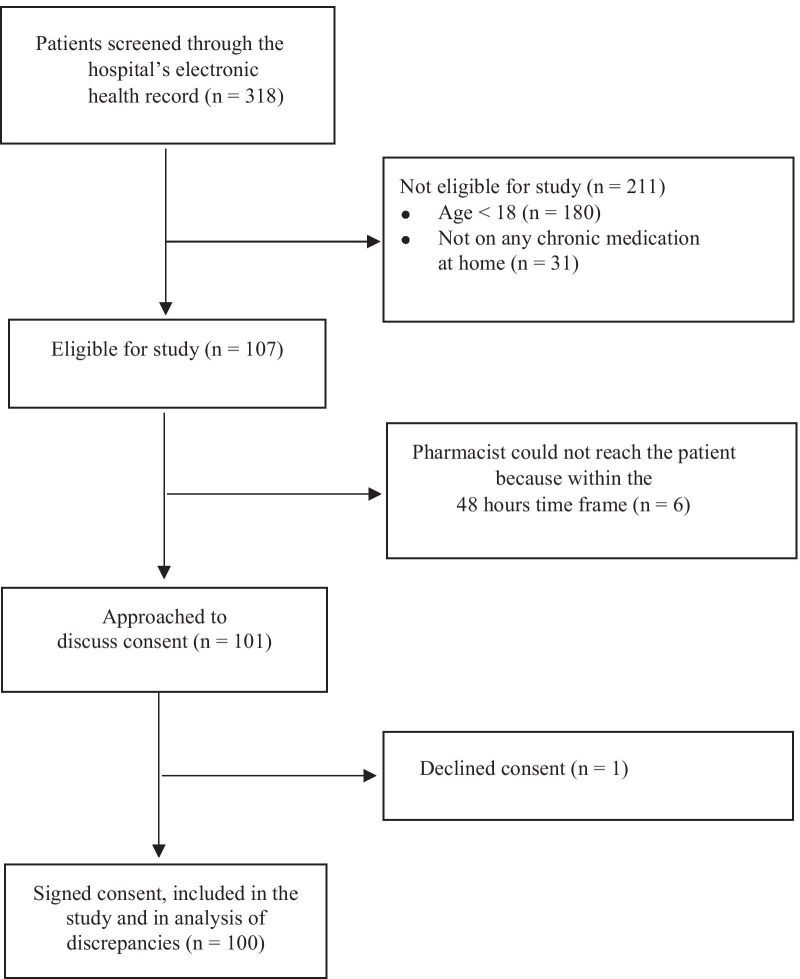
Patient enrollment flow diagram

The study included a total of 100 patients. Figure 1 shows the patient enrollment flow diagram. The patients had equal gender distribution and a mean age of 71.9 years. Almost half of the included patients took ≥ 5 home medications. Approximately 14% of patients reported having a history of allergy to ≥ 1 medication. Sociodemographic and baseline characteristics of study participants are described in Table 1.

**Table.1:** Sociodemographic and baseline characteristics

Characteristic	Frequency/percentage *N* = 100
Gender	
Male	50
Female	50
Age (years)	
Mean	71.92
Standard deviation	13.16
Orthopedic surgery type	
Elective	54
Emergency	46
Creatinine clearance (the Cockcroft–Gault equation)[26]	
30 mL/min to < 50 mL/min	17
≥ 50 mL/min	83
Number of home medications	
1	2
2	8
3	14
4	25
≥ 5	51
Allergies	
No known drug allergy	86
Yes (to one or more medication)	14

The average time needed to obtain medication history was 8.3 min, ranging between 4 and 16 min (SD 3.39 min). Twenty-four patients were interviewed within 24 h and 76 patients were interviewed within 48 h of admission. The pharmacy resident used a combination of different information sources to complete the BPMH for each patient: patient interview (98%), caregiver/family member interview (80%), examination of home medication bottles or boxes (20%) and review of previous medical record (2%). Following the critical analysis of patient cases, 24 patient cases (24%) had no discrepancies with a complete one-to-one match, and 2% included intended discrepancies explained by the patient’s clinical condition. The pharmacy resident identified 110 unintended medication discrepancies in 74 patients, with a maximum of 3 discrepancies per patient case**.**

Most of the unintended discrepancies (83.6%) involved a prescription medication, and 16.4% involved an over-the-counter medication. The most common discrepancies consisted of medication omission (89.1%), wrong dose (4.5%) and wrong frequency (6.4%). The proximal causes leading to the discrepancies included the clinician’s lack of knowledge or familiarity with the involved medication (40.9%), physicians’ non-compliance with evidence-based recommendations (40%), patient’s forgetfulness or lack of knowledge about medications (10%) and name similarity of the involved medication with another medication (8.2%). When classifying the unintended discrepancies by route of administration, 92.7% were found to involve an oral medication. Only 3.6% of the unintended discrepancies involved an inhaled medication, 2.7% involved an ophthalmic preparation and 0.9% involved a topic preparation. The most common agents involved in unintended discrepancies consisted of antihyperlipidemic agents (25.4%). Dietary supplements comprised 16.4% of the cases. Other medication classes involved diuretics (9.1%), and medications for reflux disease (7.4%). Further details are included in Table 2.

**Table.2:** Unintended discrepancies

Variable	Frequency (percentage) *N* = 110
Unintended discrepancies by type	
Omission	98 (89.1)
Wrong frequency	7 (6.4)
Wrong dose	5 (4.5)
Wrong medication	2 (1.8)
Unintended discrepancies by medication route of administration	
Oral	102 (92.7)
Inhaled	4 (3.6)
Ophthalmic	3 (2.7)
Topical	1 (0.9)
Unintended discrepancies by class	
Dietary supplements (vitamins, minerals, herbal supplements)	18 (16.4)
Medications	
Antihyperlipidemic agents	28 (25.4)
Antihypertensive agents	14 (12.8)
Diuretics	10 (9.1)
Medications for reflux disease	8 (7.4)
Antidepressants/anxiolytics	6 (5.4)
Medications for asthma/chronic obstructive pulmonary disease	6 (5.4)
Anti-diarrheal/laxatives/antispasmodics	5 (4.6)
Antipsychotics	4 (3.6)
Anti-gout agents	4 (3.6)
Oral antidiabetic/insulin	2 (1.8)
Immunosuppressants (methotrexate and mycophenolic acid)	2 (1.8)
Thyroid replacement (levothyroxine)	2 (1.8)
Analgesics (acetaminophen)	1 (0.9)
Unintended discrepancies by WHO 1st level of ATC classification	
Cardiovascular system	52 (47.3)
Various	35 (31.8)
Nervous system	10 (9.1)
Respiratory system	6 (5.4)
Musculo-skeletal system	5 (4.6)
Antineoplastic and immunomodulating effect	2 (1.8)
High-alert medications	
Insulin (glargine; degludec)	2
Immunosuppressant (methotrexate; mycophenolic acid)	2
Proximal cause of unintended discrepancies	
Clinician lack of knowledge/familiarity with medication	45 (40.9)
Physicians non-compliance with evidence-based recommendations	44 (40)
Patient forgetfulness/lack of knowledge	11 (10)
Name similarity	9 (8.2)
Reason unidentified	1 (0.9)

Moreover, around 1.8% of the unintended discrepancies involved a high-alert medication, specifically insulin. When the investigators assessed the potential severity of all medication-related discrepancies, 81 (73.6%) were judged as clinically insignificant, (24) 21.8% were judged as clinically significant, and only 4 (3.6%) were judged to be serious. No life-threatening interventions were identified. Specific examples of reconciliation errors and their assigned level of severity are presented in Table 3.

**Table.3:** Examples of reconciliation errors (REs) detected in the medication history

RE type	RE example	Severity/clinical significance
Omission	A patient with hypertension was admitted for ankle fracture surgery. His medication (valsartan/hydrochlorothiazide 160 mg/12.5 mg PO daily) was not ordered for him upon admission	Clinically significant
Incorrect dose	A patient with hypertension was admitted for right foot capsulotomy. His medication dose (moxonidine 0.2 mg PO twice daily) was incorrectly ordered for him as moxonidine 0.3 mg PO twice daily	Clinically significant
Incorrect frequency	An elderly patient with multiple comorbidities was admitted for right hip fracture with betahistine 16 mg PO daily dose. At home, he was on betahistine 16 mg PO twice daily	Clinically insignificant
Wrong medication	A patient with diabetes mellitus was taking at home insulin glargine 16 units subcutaneously at night. Upon admission, he was incorrectly ordered insulin regular sliding scale instead of his basal regimen (insulin glargine)	Serious

Based on the unintended discrepancies found, the pharmacy resident recommended a total of 110 medication-related interventions. Among these interventions, 93 (84.5%) were accepted and 17 (15.5%) were rejected by surgical residents and fellows. The most common types of medication-related interventions included: the addition of a medication (71.9%); dose adjustment (12.7%); frequency adjustment (6.4%); suggestion of alternative brands (5.4%); and discontinuation or giving medication from home supply (3.6%).

In the multivariable analysis, the number of home medications was the only variable significantly associated with the number of unintended discrepancies (*p* < 0.001) (Table 4).

**Table.4:** Unintended discrepancies—multivariable analysis

Variable	B	95% CI	Beta	*T*	*p*
Age	0.010	− 0.002 to 0.023	0.148	1.617	0.109
Number of information sources used	− 0.153	− 0.495 to 0.189	− 0.085	− 0.889	0.376
Number of home medications	0.152	0.098 to 0.206	0.492	5.594	< 0.001

Variables with a p-value of 0.2 or less in the bivariate analysis were included in the initial model. Those include: age, number of information sources used, and number of home medications

Using a backward method, the final model only retained the number of home medications

## Discussion

In this pilot study, the authors aimed to assess the impact of pharmacist-conducted medication reconciliation performed on adult patients admitted to the orthopedic surgical department with ≥ 1 chronic medication. The pharmacy resident performing medication reconciliation identified unintended medication discrepancies in 74% of the included patients and found 110 REs. The mean age of patients was around 72 years highlighting the aging population undergoing surgery and the emerging need to carefully manage their chronic medications. In the present study, 90% of the patients were taking at least 3 chronic medications. In the multivariate analysis, the risk of REs was significantly higher with higher number of home medications (*p* < 0.001). This finding has also been reported in several previous studies [16–18].

REs were most frequently detected in cardiovascular drugs, as observed in other studies [19]. This finding can be attributed to the high prevalence of cardiovascular diseases among elderly in Lebanon [20], especially that 74.3% of the study patient population were above 65 years old.

When performing preoperative medication assessment, it may be necessary to advise the patient to stop or alter some medications before an operation to ensure that they can safely undergo anesthesia and the procedure itself. Although some medicines should be stopped (e.g., anticholinesterases, monoamine oxidase inhibitors, and anticoagulants), it is important that others be continued [21]. Examples of intentional or intended omissions identified in this study include omission of aspirin for one patient and rivaroxaban for another patient. These patients were admitted for elective total knee and hip replacement surgeries, respectively. These two omissions were justified based on recommendations from American College of Chest Physicians (ACCP) [22] and European Society of Cardiology (ESC) [23]. The first patient who was on aspirin for prevention of cardiovascular events, was instructed by his physician to stop it 5 days before being admitted for his elective surgery. The second patient was receiving rivaroxaban for atrial fibrillation and he stopped it 3 days before his scheduled surgery.

The most common type of unintended discrepancies (89.1%) involved the omission of a medication that the patient was taking before admission, which is higher than what was seen in previous studies [8, 24, 25]. This can be explained by the fact that surgery resident physicians may lack familiarity with medications outside their field of specialty, medication history taking may have been incomplete and/or inconsistent, especially that many patients were not previously hospitalized at LAUMC-RH, as such lack a previous medical record for their chronic medications. Furthermore, a consistent and reliable outpatient patient profile system does not exist in Lebanon. Omission of medications can sometimes lead to clinically significant outcomes depending on the patient’s comorbidities and type and number of omitted medications [25].

Most of the identified REs were judged as clinically insignificant. Examples include omission of antihyperlipidemic agents or drugs used to treat reflux disease [24]. On the other hand, changing the dose of diuretics, omitting a beta-blocker or omitting a long-acting muscarinic antagonist in a chronic obstructive pulmonary disease (COPD) patient were examples of identified clinically significant REs. These omission errors upon admission, can compromise the stability of the patient’s chronic conditions such as hypertension or COPD warranting a close monitoring of vital signs and oxygen requirements, respectively.

The pharmacy resident identified four serious reconciliation errors (around 3.6%). Two of them were related to the unjustified switch of insulin glargine and degludec that the patients were receiving chronically at home, to regular insulin sliding scale upon admission. According to Diabetes Canada—Clinical Practice Guidelines Expert Committee for the in-hospital management of diabetes, scheduled subcutaneous insulin administration that consists of basal, bolus (prandial) and correction (supplemental) insulin components is the preferred method for achieving and maintaining glucose control in non-critically ill hospitalized patients, including surgery patients with diabetes who are eating [26]. Bolus insulin can be withheld or reduced in people who are not eating regularly, however, basal insulin should not be withheld [26]. This approach has been shown to reduce postoperative complications, including wound infections [26]. In fact, this approach is also consistent with the American Diabetes Association recommendations for the management of hyperglycemia in hospitalized patients [27]. Therefore, keeping the patients on the insulin sliding scale only without their home basal insulin may have potentially caused undesirable fluctuations in blood sugar. The other two REs identified as serious were the omission of mycophenolic acid in one patient who had kidney transplant five years ago and the omission of weekly oral methotrexate for a patient with rheumatoid arthritis. Immunomodulator agents are considered time-critical scheduled medications, which means that delayed or early administration of more than 30 min of the pre-set administration time may cause harm or sub-therapeutic effect [28]. In fact, immunomodulatory medications omission and non-adherence is a major risk factor for acute rejection of transplanted organs and subsequent loss of allografts [29]. Additionally, non-adherence to methotrexate may affect the patient’s disease activity and the cost of therapy [30].

When analyzing the proximal causes of the identified REs, the main two reasons identified were clinician lack of knowledge/familiarity with medication and physician’s non-compliance with evidence-based recommendations.

The medical team was receptive to the interventions made by the pharmacy resident as demonstrated by the high rate of intervention acceptance (around 85%). This acceptance rate is higher than what is documented in the literature for general pharmacy recommendation acceptance rate (48 to 73%) [31–34], but similar to what is documented for medication reconciliation recommendations (78%) [31] and recommendation of clinical pharmacists on surgical wards (85%) [35]. The majority of rejected recommendations, however, were associated with omission of antihyperlipidemic agents or multivitamins that the physicians considered unnecessary during the course of hospitalization. Another potential reason for rejecting the pharmacy resident interventions was the resistance of some physicians to follow evidence-based approaches that contradict their original practice with respect to the in-hospital management of certain medical conditions, namely shifting patients from their home long-acting basal insulins to sliding scale regular insulin upon hospital admission.

Those findings impact clinical pharmacy practice and highlight the role of the pharmacist as an indispensable asset on surgery units who can improve access to drug information and to updated evidence-based recommendations [36]. The findings also indicate that pharmacist-led educational sessions for the surgical residents about the proper perioperative medication management may help reduce reconciliation errors on surgical services, and prevent potentially serious patient harm.

Additionally, the findings of this study provide insight to policy makers, especially concerning the role assigned to pharmacists in medication reconciliation. The latter has been clearly defined by the American Society of Health-System Pharmacists (ASHP) in its statement on the pharmacist’s role in medication reconciliation [37]. Following the completion of this study, the pharmacy department at LAUMC-RH developed a medication reconciliation policy upon patient admission and discharge, delineating the role of each member of the healthcare team in the implementation of this process: pharmacists, nurses and physicians. In addition to developing the policy and the BPMH form, pharmacists are leading the multidisciplinary education of all healthcare professionals involved in the process. Subsequent steps include starting the implementation, following a hospital-wide rollout strategy, by unit [12]. Similarly, and in accordance with the newly released MOPH hospitals accreditation standards mentioned earlier, more hospital leaders in Lebanon are recognizing the benefits of establishing a well-defined medication reconciliation policy, outlining a clear process for its implementation [11].

With the emerging concept of value-based healthcare, and with the ongoing harsh economic and financial crises in Lebanon, hospital administrators are urged to optimize resources [38–40]. Future research is warranted to assess the cost–benefit analysis of pharmacists’ involvement in medication reconciliation which in turn can provide justification for pharmacists to perform BPMH. Such research can address, in a hospital-specific manner, the number of harmful medication errors potentially avoided per year, versus the cost of additional pharmacist full-time equivalent (FTE) needed to provide the service [12]. These studies can capitalize on both the clinical and the economic impact of the pharmacist role in medication reconciliation.

### Limitations

The authors acknowledge the potential limitations of this study. This was a single-center, single-arm study, where formal power analysis was not conducted which may have contributed to the small sample size. Additionally, the pharmacy resident was not available over the weekend, thus patients admitted for orthopedic surgery on weekends were excluded. Furthermore, since most elective surgeries are usually admitted over the weekend, this may have affected the characteristics and number of patients included in this study and the numbers of elective surgery. The risk of detection bias could not be excluded due to the fact that the investigators used their clinical judgment on classifying the severity of identified REs and their proximal causes.

Expectedly, the coronavirus disease 2019 (COVID-19) pandemic affected the number and characteristics of the enrolled patients, as the hospital took specific measures in response to the pandemic. Firstly, LAUMC-RH cancelled all elective surgeries in all specialties as part of the hospital’s strict strategy to contain the disease. This may have impacted the number of elective surgery patients represented in the study. The hospital also required that every patient admitted for surgery undergo a polymerase chain reaction (PCR) test to rule out COVID-19 infection. Therefore, the waiting phase for the PCR results caused a delay with some patient interviews or the exclusion of others in case they exceeded the 48-h inclusion time range.

## Conclusions

Pharmacy-led medication reconciliation performed within 24–48 h of admission, considerably decreased reconciliation errors in patients admitted for orthopedic surgery. Amidst an unprecedented financial crisis hitting Lebanon and limiting the hospitals’ expenditure abilities, hospital and pharmacy leaders are called, each in their capacity, to mobilize a pharmacist-driven implementation of a highly needed well-designed medication reconciliation process.

## Supplementary Information

**Additional file 1.** Medication Reconciliation Data Collection Form. This is the form the pharmacy resident used to document the medication reconciliation process. The form includes general demographic data, surgery type, total number of home medications and their indications, history of drug allergies, and results of the critical analysis of discrepancies.

## Data Availability

The datasets used and/or analyzed during the current study are available from the corresponding author on reasonable request.

## Abbreviations

BPMH: Best Possible Medication History
REs: Reconciliation Errors
LBCTR: Lebanon Clinical Trials Registry
ADEs: Adverse Drug Events
LAUMC-RH: Lebanese American University—Rizk Hospital
MOPH: Ministry of Public Health
IRB: Institutional Review Board
MATCH: Medications at Transitions and Clinical Handoffs
ISMP: Institute of Safe Medication Practices
ATC: Anatomical Therapeutic Chemical
ACCP: American College of Chest Physicians
ESC: European Society of Cardiology
COPD: Chronic Obstructive Pulmonary Disease
ASHP: American Society of Health-system Pharmacists
FTE: Full Time Equivalent
COVID-19: Coronavirus Disease 2019
PCR: Polymerase Chain Reaction
