# Supplementary material for: Value of pharmacy services upon admission to an orthopedic surgery unit
Source: J Pharm Policy Pract. 2021 Dec 6;14:103. doi: 10.1186/s40545-021-00384-x (PMC8646011; doi:10.1186/s40545-021-00384-x)
Supplement: Supplementary file 1 — Additional file 1. Medication Reconciliation Data Collection Form. This is the form the pharmacy resident used to document the medication reconciliation process. The form includes general demographic data, surgery type, total number of home medications and their indications, history of drug allergies, and results of the critical analysis of discrepancies. [file 40545_2021_384_MOESM1_ESM.pdf]

LAU School of Pharmacy

LAU Medical Center
المركز الطبّي للجامعة اللبنانية الأميركية - مستشفى رزق
Lebanese American University Medical Center-Rizk Hospital

Medication Reconciliation Data Collection Form

Patient Demographics:

MRN #: ___ Age:___ Gender:___ Wt:__ Ht:___ CrCl:___________

Allergies: _______ Admission Date: __________ Information Source:

Number of home meds:___ Surgery type: _________________

Patient Interview ☐
Family Member ☐
Previous Medical Record ☐
Home Meds Bottles Or Boxes ☐

Time needed to collect the medication history: ___min Attending Surgeon: ____________________

(Name / Dose / Route / Frequency) (including OTC Products and Herbs)	Indication	Compliant?	Last dose taken (Time)	Should continue on admission	cont'd?	substitud?	Reason for Withhold, and verified?

Current medications order form from patient medical chart:

Indication	Drug/dose/frequency/route

Critical Analysis of Discrepancies

☐ One-to-one Match ☐ Intended, Justified Discrepancies ☐ Unintended Discrepancies

Total Number of Reconciliation Errors/Unintended Discrepancies: ____________

REs by Medication Type:

☐ Over the Counter (OTC) ☐ Presciption ☐ Herbal ☐ Allergies

Discrepancies by Error Type:

☐ Unintended Omission ☐ Wrong drug ☐ Wrong dose ☐ Wrong route ☐ Wrong frequency

☐ Duplication ☐ Drug interaction ☐ Allergy alert ☐ Wrong allergy ☐ Unjustified Omission

REs by Route of Medications involved:

☐ Oral ☐ SC ☐ Buccal ☐ Inhaled ☐ Ophthalmic

☐ IM ☐ IV ☐ Rectal ☐ Otic ☐ Topical

REs by Therapeutic/Pharmacological Class of Medications involved:

☐ Diuretic ☐ Lipid Modifying ☐ Antipsychotic ☐ Drug for acid disorders ☐ Antibiotic

☐ Beta Blocker ☐ Analgesic ☐ Oral Antidiabetic ☐ Vitamin ☐ Mineral Supplements

☐ Antiepileptic ☐ Insulin ☐ Antidepressants ☐ Antidiarrheals/Laxatives/Antispasmodics

☐ Anti-Parkinson ☐ ACE/ARB ☐ Drugs for asthma/COPD **Other. Specify:** ______________________

REs by Surgical Department:

☐ Cardiovascular ☐ Urology ☐ Orthopedics ☐ Gastrointestinal ☐ Neurology ☐ Plastic ☐ Thoracic

REs Involving High-Alert Medication ☐ Yes ☐ No

Proximal cause leading to the reconciliation errors (as determined by the pharmacist)

☐ Clinician knowledge (ie, lacking familiarity with medication regimens)

☐ Patient knowledge (ie, lacking familiarity regarding their medication regimens)

☐ Dosage form confusion

☐ Brand name and generic name similarity (Look-Alike and Sound-Alike)

☐ Non-compliance of physicians with evidence-based recommendations

☐ Unknown (ie, pharmacist unable to determine potential cause leading to medication error)

Potential severity of the error:

☐ Clinically insignificant (Error that would not likely cause harm)

Ex: Omission of oral ferrous sulfate, omission of statin, wrong PPI Dose

☐ Significant (have the potential to cause harm and require increased monitoring)

Ex: Wrong dose of antihypertensive

☐ Serious (have the potential to cause harm and likely to require 1)intervention or 2)prolonged hospital stay)

Ex: Wrong dose of warfarin, omission of levertiracetam for seizure disorder

☐ Life-threatening (have the potential to cause death)

Ex: Wrong controlled release morphine death, Nifedipine 90mg immediate release ordered instead of SR.

Medication-related Interventions:

Type of intervention:

☐ Adjust dose ☐ Add a drug ☐ D/C a drug ☐ Adjust frequency ☐ Adjust route

☐ Educate the patient ☐ Highlight drug interactions ☐ Highlight a drug-disease interactions

☐ Monitor drug parameter(s) ☐ Discontinue drug/give from home supply ☐ Suggesting alternative brand

Total number of interventions: ________________________________

☐ Accepted ☐ Rejected ☐ Pending review

Intervention type	Intervention	Accepted
